# Pharmacogenomic Pathways Underlying Variable Vedolizumab Response in Crohn’s Disease Patients: A Rare-Variant Analysis

**DOI:** 10.3390/biomedicines14010203

**Published:** 2026-01-17

**Authors:** Biljana Stankovic, Mihajlo Stasuk, Vladimir Gasic, Bojan Ristivojevic, Ivana Grubisa, Branka Zukic, Aleksandar Toplicanin, Olgica Latinovic Bosnjak, Brigita Smolovic, Srdjan Markovic, Aleksandra Sokic Milutinovic, Sonja Pavlovic

**Affiliations:** 1 Institute of Molecular Genetics and Genetic Engineering, University of Belgrade, 11042 Belgrade, Serbia; biljana.stankovic@imgge.bg.ac.rs (B.S.); mihajlo.stasuk@gmail.com (M.S.); vladimir.gasic@imgge.bg.ac.rs (V.G.); bojan.ristivojevic@imgge.bg.ac.rs (B.R.); ivana.grubisa@imgge.bg.ac.rs (I.G.); branka.zukic@imgge.bg.ac.rs (B.Z.); 2 Clinic for Gastroenterohepatology, University Clinical Center of Serbia, 11000 Belgrade, Serbia; aleksandartoplicanin4@gmail.com (A.T.); asokicmilutinovic@gmail.com (A.S.M.); 3 Clinic for Gastroenterology and Hepatology, University Clinical Center of Vojvodina, 21000 Novi Sad, Serbia; olgica.latinovic@gmail.com; 4 Internal Clinic, Department of Gastroenterohepatology, Clinical Center of Montenegro, 81000 Podgorica, Montenegro; brigita.smolovic@kccg.me; 5 Faculty of Medicine, University of Montenegro, 81000 Podgorica, Montenegro; 6 School of Medicine, University of Belgrade, 11000 Belgrade, Serbia; srdjanmarkovic@yahoo.com; 7 Department of Gastroenterology and Hepatology, University Hospital Medical Center “Zvezdara”, 11120 Belgrade, Serbia

**Keywords:** Crohn’s disease, gene-set burden analysis, pharmacogenomics, rare variants, vedolizumab

## Abstract

**Background/Objectives:** Vedolizumab (VDZ), a monoclonal antibody targeting α4β7 integrin, is used in Crohn’s disease (CD) management, yet patients’ responses vary, underscoring the need for pharmacogenomic (PGx) markers. This study aimed to identify PGx pathways associated with suboptimal VDZ response using a rare-variant analytical framework. **Methods:** DNA from 63 CD patients treated with VDZ as first-line advanced therapy underwent whole-exome sequencing. Clinical response at week 14 classified patients as optimal responders (ORs) or suboptimal responders (SRs). Sequencing data were processed using GATK Best Practices, annotated with variant effect predictors, and filtered for rare damaging variants (damaging missense and high-confidence loss-of-function; minor allele frequency < 0.05). Variants were mapped to genes specific for SRs and ORs, and analyzed for pathway enrichment using the Reactome database. Rare-variant burden and composition differences were assessed with Fisher’s exact test and SKAT-O gene-set association analysis. **Results:** Suboptimal VDZ response was associated with pathways related to membrane transport (ABC-family proteins, ion channels), L1–ankyrin interactions, and bile acid recycling, while optimal response was associated with pathways involving MET signaling. SKAT-O identified lipid metabolism-related pathways as significantly different—SRs harbored variants in pro-inflammatory lipid signaling and immune cell trafficking genes (e.g., *PIK3CG*, *CYP4F2*, *PLA2R1*), whereas ORs carried variants in fatty acid oxidation and detoxification genes (e.g., *ACADM*, *CYP1A1*, *ALDH3A2*, *DECR1*, *MMUT*). **Conclusions:** This study underscores the potential of exome-based rare-variant analysis to stratify CD patients and guide precision medicine approaches. The identified genes and pathways are potential PGx markers for CD patients treated with VDZ.

## 1. Introduction

Crohn’s disease (CD) is a disorder that belongs to inflammatory bowel diseases (IBDs). CD results from a dysregulated interaction between genetic susceptibility, impaired intestinal epithelial barrier function, alterations in gut microbiota, and aberrant mucosal immune responses. Genetic variants affecting innate immune sensing, autophagy, and adaptive immune regulation predispose to an exaggerated immune response against luminal antigens, leading to persistent activation of antigen-presenting cells and effector T-cell pathways, particularly Th1 and Th17 axes. This sustained immune activation promotes transmural intestinal inflammation and tissue damage [1]. The incidence of CD in Western and Northern Europe is high (ranging from 9.1 to 17.5 per 100 000 person-years), but with a stabilizing rate, while a significant rise in CD has been detected in Eastern Europe. The prevalence of CD is constantly increasing, since it is a chronic incurable disease and an increase in its onset in young age has been observed [2].

The management of CD is extremely complex, because its clinical expression is heterogeneous and the course of the disease is diverse. Each patient requires a specific therapeutic protocol. The backbone of the CD therapy consists of corticosteroids, immunosuppressants, and immunomodulators. Besides those therapeutics used earlier in the disease course, biological treatment could be useful in the presence of an incomplete clinical response. The introduction of monoclonal antibodies (mAbs) designed to inhibit the tumor necrosis factor-α (TNF-α) inflammatory pathway (e.g., infliximab (IFX), adalimumab (ADL), golimumab (GOL), and certolizumab pegol (CLZ)) brought a key improvement in CD treatment [3]. Although the use of these therapeutics leads to an improvement of the disease course and reduction in the risk of surgery, 30% of patients experience a primary non-response to anti-TNF therapies and, over time, around 50% of patients have to stop the treatment due to secondary non-response or the presence of adverse effects [4]. Therefore, other mABs have been introduced in the therapeutic protocols for CD, such as vedolizumab (VDZ), a monoclonal antibody to the α4β7 integrin, and Ustekinumab (UST), which inhibits the p40 subunit of IL-12 and IL-23 [5]. However, similarly to the response to other therapies, it is shown that success in achieving persistent clinical remission with these new agents has been variable in CD patients [6,7].

A loss of response and adverse effects of therapies for CD could be a consequence of different mechanisms, both non genetic (pharmacokinetics, pharmacodynamics, patient’s microbiome) and genetic (a unique profile of the patient) [8,9]. Inconsistency in response to various therapeutics made it necessary to take those mechanisms into consideration in CD therapeutic protocols. Pharmacogenomics (PGx), a main cornerstone of precision medicine, has caught the attention of clinicians involved in the treatment of CD patients. In order to provide the most efficient and the least harmful (toxic) treatment protocol to each patient, they have an intention to introduce PGx markers in everyday practice. Methodological progress, such as high-throughput genomics analysis, has contributed mostly to the knowledge of relevant PGx markers to be considered in the treatment of CD patients.

There are well established PGx markers which are considered during CD treatment [10,11,12]. The ones with the most confirmation studies are variants in the *TPMT* gene which are recommended for thiopurine drugs’ pretreatment testing by the U.S. Food and Drug Administration [13,14]. Additionally, great efforts have been made to identify genetic markers that may predict individual response to anti-TNF therapy [15].

Even with maximum research endeavors, a personalized therapeutic strategy in CD patients was not achieved [9,16,17]. Therefore, the need for expanded investigations of VDZ PGx has to be emphasized, since there is a lack of systematic study in the field of VDZ PGx. There were studies in which pharmacogenetic determinants of anti-TNFs drugs were tested as PGx markers for VDZ, but no correlation was found [18]. There was also a study in which it was shown that the HLA-DQA1*05 allele (rs2097432) was not associated with increased immunogenicity for VDZ [19]. Vedolizumab is a gut-selective α4β7 integrin antagonist that blocks adhesion of memory T-cells to the mucosal addressin cell adhesion molecule-1 (MAdCAM-1). That is how VDZ reduces the infiltration of these inflammatory cells into gut mucosal tissue and suppresses gut inflammation. MAdCAM-1 is a protein that in humans is encoded by the *MADCAM1* gene [20,21]. Even the *MADCAM1* gene has not been investigated as a potential pharmacogene.

This is a pioneer study in the field of VDZ pharmacogenomics. In order to overcome the knowledge gap regarding genetic markers of poor VDZ response, we have integrated whole-exome sequencing with a rare-variant analytical framework, combining pathway-level enrichment with carrier burden and variant composition analyses. This strategy allows us to assess whether rare damaging variants within specific biological pathways are associated with treatment outcome, providing new insight into how genetic background may influence response to VDZ.

## 2. Materials and Methods

### 2.1. Study Group

A cohort of 63 patients diagnosed with CD and treated with VDZ as a first-line advanced therapy, who completed the 14-week induction period, was recruited between June 2023 and February 2025 at the Clinic for Gastroenterohepatology, University Clinical Center of Serbia, Department of Gastroenterology and Hepatology, University Clinical Center of Vojvodina and the Department of Gastroenterology and Hepatology, University Hospital Medical Center “Zvezdara”. Inclusion criteria were age ≥18 years and signed informed consent. Exclusion criteria included inability to understand and sign informed consent due to cognitive deficit or language misunderstanding, and prior treatment with any other biologic agent or targeted small molecule therapy for CD.

VDZ was administered as an intravenous infusion (300 mg) at weeks 0, 2, 6, and 14 during the induction phase. In some patients, an additional infusion was given at week 10 based on clinical indication. After 14 weeks of treatment, clinical and biomarker outcomes were assessed. Patients were categorized into two groups: optimal responders (ORs), defined as those who achieved clinical remission, and suboptimal responders (SRs), defined as those who did not. Clinical remission was defined as the absence of diarrhea and abdominal pain at week 14, based on physician assessment and patient-reported symptoms [22]. In all patients, changes in biomarker levels, including C-reactive protein (CRP) and fecal calprotectin, were evaluated at week 14. For the purpose of this study, blood samples were collected from CD patients for DNA isolation and further whole-exome sequencing (WES) analysis.

The study was conducted in accordance with the ethical standards of the Declaration of Helsinki, and approved by the Ethics Board, Clinical Centre of Serbia, on 28 February 2020, Prot. No. 432/7 and on 13 October 2025, Prot. No. 1600/31. Ethical approvals were also obtained from the Ethics Board of University Clinical Center of Vojvodina (23 October 2025, Prot. No. 00-378) and the Ethics Board of University Hospital Medical Center “Zvezdara” (7 November 2025, Prot. No. 05/11/2025). Written informed consent was obtained from all participants prior to inclusion in the study.

### 2.2. Statistical Analysis of Demographic and Clinical Characteristics

Statistical analyses were performed to compare demographic and clinical characteristics between SRs and ORs. Continuous variables were summarized using the median and interquartile range or minimum–maximum values, and group differences were assessed using the non-parametric Mann–Whitney U test for unpaired and Wilcoxon signed-rank test for paired data. Categorical variables were presented as counts and percentages, and differences between groups were evaluated using Fisher’s exact test. A *p*-value < 0.05 was considered statistically significant. Statistical analyses were conducted using R software v4.4.2.

### 2.3. Whole-Exome Sequencing and Data Processing

We performed the whole-exome sequencing using DNA Prep with an Exome 2.5 Enrichment kit (Illumina, San Diego, CA, USA) on an Illumina NextSeq2000 platform, following manufacturer recommendations. The goal was to generate high-quality sequencing data and call genetic variants with confidence before downstream analysis.

For processing WES data, GATK Best Practices workflow was used [23]. The workflow comprised two main phases: (1) mapping—raw FASTQ data were aligned to the hg38 reference genome using BWA, followed by flagging duplicates (GATK v4.6.0. MarkDuplicates) and performing Base Recalibration (GATK BaseRecalibrator and ApplyBQSR) and (2), variant calling (GATK HaplotypeCaller). Variant calling was performed for each sample, creating a gVCF file, then single-sample gVCF files were merged into a multi-joined gVCF file (GATK CombineGVCFs) and subsequently genotyped (GATK GenotypeGVCFs), creating a raw cohort (c)VCF file.

The raw cVCF was further filtered in order to retain variants with high-quality individual genotypes using bcftools v1.17. For this purpose, individual genotypes were set to “missing” if they had low coverage (sequencing depth (DP) < 20 reads), genotype quality (GQ) < 20, and allelic balance (AB) in heterozygous carriers < 0.2 [24]. These filters ensure that only variants with reliable sequencing support are retained, minimizing false-positive calls. Variants absent in more than 5% of samples were excluded. Remaining variants were left-normalized and transformed into biallelic using the “bcftools norm-m-both” command.

### 2.4. Variant Annotation

Next, the cVCF file was annotated using the Variant Effect Predictor (VEP) version 113 to translate raw genetic calls into functional information.

Variants were annotated on a per-gene, per-allele basis using the “pick_allele_gene” option to ensure that each annotation corresponded to a specific allele within a single gene. Annotations were assessed by selecting canonical transcripts. In case more than one canonical transcript was available, the one with the most severe consequence was selected. For predicting the functional effect of genetic variants, several annotation resources were used: (a) for missense variants, dbNSFP v5.1 plugin was used to assess REVEL and MetaRNN prediction scores; (b) LoFtee plugin (https://github.com/konradjk/loftee, accessed 1 March 2025) was used to predict effect of frameshift, start lost, stop lost, and stop gained variants, as well as essential splice variants (splice donor and acceptor variants). After the annotated file was created, data were further analyzed inside the jupyter notebook script. If a variant was assigned with two consequences, a more severe consequence was taken into account (for example, missense_variant&splice_region_variant > missense_variant). For making a more robust judgment about missense variant predicted deleteriousness, consensus scoring was performed by averaging REVEL and metaRNN scores, which is important in case single-tool predictions disagree or are missing. Variants assigned as high confidence by Loftee were predicted as loss-of-function (pLoF) variants. Rare damaging variants with minor allele frequency (MAF) < 0.05 found in either gnomAD exomes or gnomAD genomes (v3.1.2, all populations) were selected for mapping candidate genes potentially associated with suboptimal VDZ response and further enrichment analysis.

### 2.5. Enrichment Analysis

Enrichment analysis was performed to identify biological pathways disproportionately affected by rare variants with predicted damaging effects (damaging missense and pLoF) in each patients’ response group.

Variants were mapped to their associated genes, identifying affected gene-sets in the SR and OR groups. Over-representation analysis (ORA) on identified gene-sets was performed separately for each response group using the Reactome Human Pathways 2024 database via the GSEApy python library module Enrichr v1.1.8 [25,26]. When a gene belonged to multiple overlapping pathways, it was included in each relevant pathway to capture all potential functional associations. Redundancies among gene-sets were addressed by using adjusted *p*-values from the ORA, while results were interpreted considering the Reactome parent–child pathway hierarchy. Results were scored using Enrichr’s combined score, which integrates the adjusted *p*-value from Fisher’s exact test with a z-score reflecting deviation from expected rank. A higher combined score generally indicates a more significant and relevant enrichment. For each pathway, the combined score difference between groups (ΔCombined Score = OR − SR) was computed, representing relative pathway activation. In parallel, the minimum adjusted *p*-value < 0.05 across both groups was used as a measure of statistical support. These values were visualized using a volcano plot, where the x-axis represents the enrichment score difference between groups, and the y-axis represents the negative log10 of the minimum adjusted *p*-value. Pathways with the greatest enrichment divergence and statistical significance were highlighted.

### 2.6. Rare-Variant Pathway Association Analysis

Because enrichment analysis summarizes variant counts at the gene-set level and does not incorporate which individuals carry each variant, it cannot statistically test group differences. Therefore, we additionally applied carrier burden and SKAT-O analysis, which uses individual-level genotype data to formally test whether rare variants within a pathway differ in frequency or variant composition between SR and OR groups.

In carrier burden analysis, the number of individuals carrying at least one rare damaging variant for each enriched pathway was quantified and compared between SR and OR groups using Fisher’s exact test.

Additionally, a gene-set based rare-variant association analysis was performed using the SKAT-O (optimal sequence kernel association test) [27,28], implemented in the SKAT R package (https://cran.r-project.org/web/packages/SKAT/index.html, accessed 15 May 2025). This test combines the burden and variance-component models to optimally detect both unidirectional and bidirectional effects of rare variants. Gene-set variant effects were evaluated per pathway. Covariates such as age and sex were included in the SKAT-O null model. Multiple testing correction was applied using the Benjamini–Hochberg false discovery rate (FDR) procedure, using an FDR threshold of 0.05, allowing 5% of false discoveries among reported findings.

## 3. Results

### 3.1. Cohort Description

A total of 63 individuals with CD were included in the study. Of these, 31 patients exhibited a suboptimal response to VDZ, while 32 demonstrated an optimal response. The age distribution was comparable between the SR and OR groups. Similarly, there were no significant differences in the gender distribution between the two groups.

No statistically significant differences were observed in disease phenotypes or in the use of immunomodulatory therapies (azathioprine and methotrexate) between SR and OR patients. When fecal calprotectin was compared between baseline and 14th week, a significant decrease was detected at week 14 compared to baseline, in both the SR and OR groups (*p* = 0.007, *p* = 0.001, respectively) (Figure S1). Although fecal calprotectin levels were higher in the SR compared to the OR group, both at baseline and at week 14, the differences between groups did not reach statistical significance (*p* = 0.11, *p* = 0.14, respectively). Comparing CRP levels between baseline and week 14, there were no statistically significant differences between these two time points in both the SR and OR groups (*p* = 0.224, *p* = 0.951, respectively). However, CRP levels were significantly higher in the SR group compared to the OR group at both baseline and week 14 of therapy (*p* = 0.01 and *p* = 0.04, respectively) (Figure S2). There was no statistically significant difference in the number of patients that received additional infusion of VDZ at week 10 between SR and OR (Table 1).

### 3.2. Genetic Variant Detection

We obtained a total of 132,887 genetic variants in raw cVCF. After quality control and filtering, 24,093 variants remained. Following the selection of damaging missense variants, defined by a mean value of REVEL and MetaRNN score > 0.5, and high-confidence pLoF variants, the number of retained variants was reduced to 1639. Among these, 1268 were classified as rare (MAF < 5% in gnomAD). The distribution of variants between SRs and ORs is presented in Table 2.

### 3.3. Enrichment Analysis

**Figure 1:**
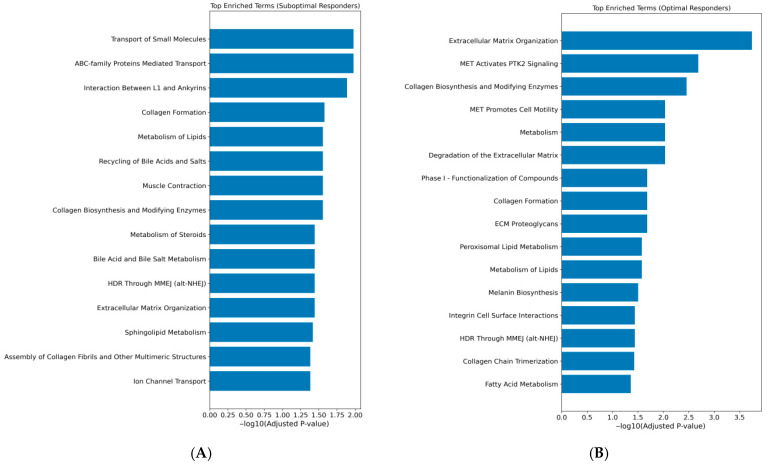
Reactome pathways significantly enriched with gene-sets associated with (**A**) suboptimal and (**B**) optimal VDZ response in CD patients. Gene-sets in each group were defined as sets of genes harboring rare damaging variants The x-axis shows −log10(adjusted *p*-values), where a value of 1.3 indicates the cutoff for statistical significance.

After mapping rare damaging variants to their corresponding genes, we identified gene-sets associated with suboptimal (*n* = 607) and optimal (*n* = 644) response to VDZ treatment. Over-representation analysis was performed with identified gene-sets using the Reactome Human Pathway 2024 to characterize pathway-level differences between two groups of patients. The analysis revealed distinct pathway enrichment profiles between the SRs and ORs (Figure 1, Tables S1 and S2).

In the SR group, 15 pathways were significantly enriched, while the OR group exhibited enrichment in 16 pathways (Figure 1). Both groups showed enrichment in pathways related to ECM organization. However, the OR group displayed a broader enrichment of ECM-associated pathways, also including those involving proteoglycans and integrin-mediated cell surface interactions. Lipid metabolism pathways were also represented in both groups, but with distinct profiles. In the SR group, the enrichment was driven by transmembrane lipid transport, such as the “Recycling of Bile Acids and Salts” pathway (adjusted *p* = 0.028), whereas the OR group showed enrichment in intracellular lipid processing, specifically the “Peroxisomal Lipid Metabolism” pathway (adjusted *p* = 0.027) (Figure 1, Table S3).

Unique significantly enriched pathways in the SR group were “ABC-family Proteins Mediated Transport” (adjusted *p* = 0.011), “Ion Channel Transport” (adjusted *p* = 0.041) and “Interactions between L1 and ankyrins” (adjusted *p* = 0.013) (Table S3). In contrast, the OR group uniquely showed significant enrichment in signal transduction pathways, particularly those involving MET signaling, “MET Activates PTK2 Signaling” and “MET Promotes Cell Motility” (adjusted *p* = 0.002, *p* = 0.009, respectively) (Table S3).

**Figure 2:**
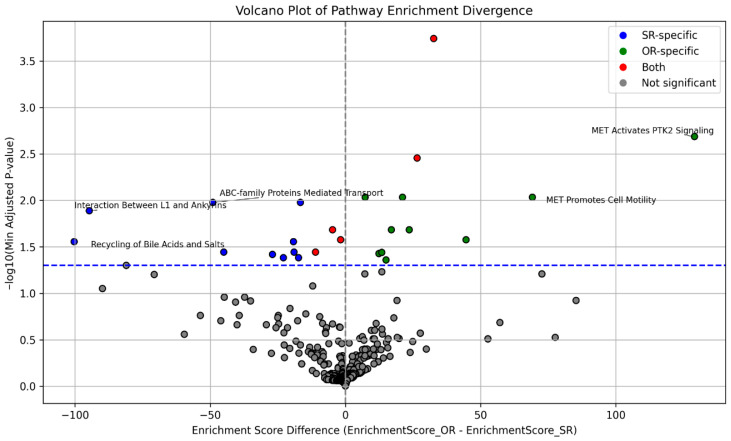
Volcano plot showing differential pathway enrichment between OR and SR groups. Differential pathway enrichment was calculated using difference in enrichment scores (Enrichr’s combined score) between OR and SR groups using the following formula: enrichment score difference = enrichment score_OR—enrichment score_SR, and presented on x-axis. A larger value of enrichment score difference indicates pathways specific for ORs (positive x-axis values) or SRs (negative x-axis values). The y-axis shows results of the enrichment analysis test and it is represented as −log_10_ of lower p-adjusted value obtained either for OR or SR groups. Blue dashed line represents cutoff for statistical significance (p-adjusted = 0.05). Pathways are colored by significance (insignificant enrichments are in gray) and response group specificity (pathways significantly enriched only in the SR group are in blue, significantly enriched only for ORs are in green, while significantly enriched in both groups are in red). Labeled points with pathway names represent the five most divergent pathways identified by enrichment score and significance. ORs—optimal responders; SRs –suboptimal responders.

Additionally, a comparative pathway enrichment analysis was conducted to highlight the most pronounced differences between the SR and OR groups. This analysis quantified how strongly each pathway was enriched in one group relative to the other by calculating the difference in Enrichr’s combined enrichment scores. To help interpretation, these results are visualized in a volcano plot (Figure 2), where points further to the right indicate pathways more impacted by rare damaging variants in ORs, and points further to the left indicate stronger enrichment in SRs. Five pathways with the largest separation between groups were annotated on the plot to highlight those showing the strongest divergence. Taken together, this comparison illustrates that SR and OR patients differ not only in which pathways are enriched, but also in how strongly these pathways are impacted, pointing to distinct biological processes that may influence VDZ response.

### 3.4. Rare-Variant Pathway Burden Analysis

We quantified the number of individuals carrying at least one rare damaging variant per enriched pathway and compared carrier frequencies between groups. Carriers of damaging variants in the “Ion Channel Transport” pathway were significantly more prevalent in the SR group (58%) than in the OR group (28%) (odds ratio = 3.5, *p* = 0.02). A similar phenomenon was observed for the “Interaction Between L1 and Ankyrins”, with 26% of SR patients carrying damaging variants versus 6% in ORs (odds ratio = 5.2, *p* = 0.04). No significant differences in carrier burden were observed for other pathways (Table S4).

Next, SKAT-O was used to assess whether SR and OR patients differ in how rare damaging variants are distributed within each enriched pathway. SKAT-O was used in addition to the carrier burden test because it can detect more subtle genetic differences, including situations where variants may have mixed or opposing effects, that may not appear when only counting how many participants carry a variant. SKAT-O revealed five pathways that significantly differed between SRs and ORs—“Metabolism” (*p* = 0.013), “Metabolism of Lipids” (*p* = 0.0007), “Fatty Acid Metabolism” (*p* = 0.011), “Metabolism of Steroids” (*p* = 0.04), and “Phase I Functionalization of Compounds” (*p* = 0.022) (Table S5). Notably, all five pathways are nested under the overarching “Metabolism” parent pathway, with three—“Metabolism of Lipids”, “Fatty Acid Metabolism”, and “Metabolism of Steroids”—further grouped under the “Metabolism of Lipids” branch, pointing toward lipid-related metabolic processes as a key differentiating feature. After FDR correction for multiple testing, only “Metabolism of Lipids” remained statistically supported, underscoring the strength of its association with variability in VDZ response.

Together, the results highlight complementary modes of pathway involvement: some driven by the quantity of damaging variants or the quantity of pathway-involved genes, and others by their qualitative configuration, emphasizing the value of integrating multiple rare-variant association approaches.

### 3.5. Genes Related to Differential VDZ Response

To further visualize the distribution of variants in genes across pathways and individuals, we constructed two binary heatmaps. The first heatmap (Figure S3) included pathways that were identified as being associated with SRs in enrichment and carrier burden analyses (“Ion Channel Transport”, “ABC-family Proteins Mediated Transport”, “Recycling of Bile Acids and Salts”, “Interaction Between L1 and Ankyrins”). Patterns in the panel show a higher number of pathway-involved variants in SRs compared to ORs as the primary driver of association. Additionally, in Table S6 [29,30,31,32,33,34,35,36,37,38,39,40,41,42,43,44,45,46,47,48,49,50,51,52,53,54,55,56,57,58,59,60,61,62,63,64,65], we describe genes from these pathways among SR patients and their previous relation with human and/or mouse models of IBD, IBD-related co-morbidities or inflammatory processes associated with the intestine.

A number of genes involved in ion transport (*CFTR*, *CLCA4*, *TRPM2*, *TRPV5*) were already studied in IBD, underscoring the role of disrupted mucosal hydration, epithelial stress, and barrier dysfunction in sustaining inflammation [29,33,35,50]. Additionally, ATPases (*ATP8A2*, *ATP11A/B*) implicated in membrane homeostasis and intracellular ion regulation were also previously associated with IBD [30,31,32], suggesting defects in epithelial polarity, vesicle trafficking, and calcium signaling as mechanisms of disease development. We identified a prominent group of ABC transporters as well (*ABCA*, *ABCB*, *ABCC* subfamilies) and solute carriers (*SLC10A2*, *SLC51A*, *SLCO1B3*), associated with lipid efflux and bile acid recycling. These transporters were associated with IBD and/or primary sclerosing cholangitis (PSC), a condition commonly connected with IBD [42,43,44,46,58,59,66]. In addition, genes encoding voltage-gated sodium channels (*SCN9A*, *SCN10A*), non-selective cation channel (*TRPA1*), and adhesion-related genes (*NFASC*, *SPTA1*) suggest a contribution of neuroimmune activation and visceral hypersensitivity to disease persistence, also previously found relevant in IBD [34,60,61].

The second heatmap (Figure S4) displays pathways identified as significant in the SKAT-O analysis. In Table S7 [44,46,48,49,66,67,68,69,70,71,72,73,74,75,76,77,78,79,80,81,82,83,84,85,86,87,88,89,90,91,92,93,94,95,96,97,98,99,100,101,102,103,104,105,106,107], we present genes from the “Metabolism of Lipids” as the most profound pathway found to be affected in SRs and their previous association with intestinal inflammation, IBD, and related co-morbidities. Despite harboring damaging variants in both groups, the functional categorization of altered genes diverges significantly (Figure S4). ORs predominantly carried variants in genes involved in metabolic support and detoxification, such as *ACADM*, *ACOXL*, *DECR1/2*, *MMUT* (fatty acid β-oxidation) [108,109,110], *CYP1A1*, *CYP1B1*, *CYP2C9*, *ABCG2*, and *ALDH3A2* (xenobiotic metabolism) [90,111,112,113,114], important for epithelial energy balance and barrier integrity. In contrast, SRs carried variants in genes linked to immune activation and inflammatory lipid signaling, such as *PIK3CG* (key in leukocyte chemotaxis and integrin signaling) [82,83,84,85], *CYP4F2*, *PLA2R1*, *SPTLC3* (eicosanoid and sphingolipid pathways) [69,103,106], and bile acid/steroid metabolism genes (*SLC10A2*, *SLC51A*, *CYP11B2*, *HSD3B2*) [67,70,76,93,94,115], previously implicated in CD-associated bile acid malabsorption and mucosal damage.

## 4. Discussion

This study provides new insights into the genetic underpinnings of variable responses to VDZ therapy in patients with CD, emphasizing the role of rare damaging genetic variants in shaping individual treatment outcomes. By integrating WES data with pathway enrichment and gene-set burden analyses, we uncovered distinct molecular signatures in patients with suboptimal and optimal VDZ response, suggesting that specific genetic architectures may predispose patients to differential therapeutic efficacy.

In our study, SRs were characterized by enrichment in pathways related to membrane transport (ABC-family proteins, ion channels), L1CAM–ankyrin interactions, and bile acid recycling. ABC transporters are crucial for gut homeostasis, as they regulate the efflux of bile acids, xenobiotics, and lipids in the gut mucosa, maintaining epithelial barrier integrity and controlling luminal toxicity [116]. Dysregulation of genetic variants in these transporters’ genes can lead to epithelial stress, impaired bile acid detoxification, and dysregulated lipid handling, contributing to intestinal inflammation and IBD development [47,116,117].

Also, alterations in bile acid (BA) metabolism are increasingly recognized as contributors to IBD pathogenesis. Inflammation and dysbiosis in IBD disrupt the microbial conversion of primary to secondary BAs, leading to a decrease in anti-inflammatory secondary BAs and impaired activation of regulatory receptors such as FXR and TGR5. This imbalance promotes mucosal inflammation, epithelial barrier dysfunction, and aberrant immune responses. Additionally, the accumulation of toxic primary BAs may further aggravate epithelial damage, establishing a pro-inflammatory loop that perpetuates intestinal injury in IBD patients [55,70].

Additionally, we found a number of genes involved in nociception and pain sensitivity in SRs (*TRPA1* and *TRPV5*, as well as *SCN9A* and *SCN10A*), that have been related to IBD [33,34,61,62]. Anti-nociceptive function and diminished abdominal pain perception in IBD is an important consideration, since those patients were less likely to be able to seek medical attention on time and were more likely to develop complications. These pathways may contribute to sustained inflammation, which overrides the therapeutic effects of VDZ.

In contrast, OR patients exhibited stronger enrichment in pathways within the MET–PTK2 signaling axis. c-Met is a receptor tyrosine kinase which, upon activation by its ligand, the hepatocyte growth factor (HGF), mediates many important signaling pathways that regulate cellular functions such as survival, proliferation, and migration [118]. PTK2, a downstream effector of MET and integrins, regulates cytoskeletal remodeling, epithelial adhesion, and immune cell trafficking, functions often dysregulated in IBD. c-MET and integrin cooperation can function in both ways, particularly, c-Met upon HGF stimulation promotes integrin activity which can in turn bind ECM ligands and induce cellular responses such as adhesion and migration. In a similar manner, integrin activation, through binding to its ligand in the ECM, leads to downstream c-Met phosphorylation, enabling c-Met-induced intracellular signaling and diverse cellular functions [118]. Disruption of MET signaling has been shown to reduce inflammation in colitis models, underscoring its pro-inflammatory role [119]. Also, deletion of c-MET in immune cells in a bleomycin-induced pulmonary fibrosis mouse model led to an earlier but controlled recruitment of T-cells, macrophages, and neutrophils, resulting in reduced inflammation [120]. The presence of damaging variants in MET–PTK2-related genes in OR patients may impair these inflammatory and migratory signals, resulting in reduced immune cell recruitment and a tissue environment more permissive to mucosal healing. In this context, the VDZ mechanism of blocking lymphocyte trafficking may act synergistically with altered signaling to support treatment efficacy.

In our analysis, both SR and OR groups demonstrated significant enrichment of pathways related to extracellular matrix (ECM) organization, with ECM organization emerging as the top enriched pathway in the OR group. The ECM is now understood as an active regulator of inflammation in IBD, shaping leukocyte adhesion, migration, and retention through biochemical and structural interactions. Prior transcriptomic work has shown that pre-treatment biopsies from VDZ non-responders exhibit a significantly higher expression of stromal and fibroblast-derived ECM programs, spatially confined to inflamed mucosa. This stromal-dominant tissue state appears to sustain inflammation independently of α4β7^+^ lymphocyte trafficking, offering a mechanistic explanation for the reduced efficacy of VDZ in these individuals [121]. Consistently, another study reported elevated serum biomarkers of ECM formation and degradation in patients who discontinued VDZ within 12 months, compared with long-term VDZ users, supporting ECM remodeling as a biological state associated with non-response [122]. Our study approached the ECM from a complementary angle, identifying rare, damaging DNA variants in ECM-related genes. Rather than reflecting increased ECM activity, these variants may impair ECM-mediated inflammatory signaling that contribute to resistance to VDZ. Thus, in contrast to prior work showing upregulated ECM programs in non-responders, the presence of ECM-disrupting genetic variants in our OR cohort may help explain why these patients demonstrated more efficient therapeutic outcomes. However, in our study, the involvement of ECM is not exclusively related only to the OR group, implicating more complex VDZ response mechanisms.

The SKAT-O gene-set association analysis additionally revealed differences in lipid metabolism-related pathways between the SR and OR groups. In SR patients, rare damaging variants were found in genes involved in pro-inflammatory lipid signaling and immune cell trafficking (e.g., *PIK3CG*, *CYP4F2*, *PLA2R1*), potentially sustaining inflammatory circuits that counteract VDZ’s anti-integrin mechanism. In contrast, OR patients exhibited damaging variants in genes related to fatty acid oxidation and detoxification (e.g., *ACADM*, *CYP1A1*, *ALDH3A2*, *DECR1*, *MMUT*), suggesting that these metabolic disruptions may shift immune-epithelial homeostasis in a direction more compatible with VDZ efficacy.

Alterations in lipid and lipid metabolite profiles in IBD patients were subjected to multiple studies. Compared to the healthy population, IBD patients are characterized by lower total cholesterol and higher triglyceride serum levels. This tends to be more prominent in CD compared to UC patients [123]. Lipid composition of the colon’s mucosal lining is also altered in IBD patients. Treatment naive UC patient samples show a distinct lipid milieu, characterized by elevated levels of phosphatidylcholines, ceramides, and sphingomyelines [124]. Also, the peroxisomal lipid metabolism and oxidative stress regulation pathways contribute to an altered intestinal epithelial structure in IBD [125]. Lipidomics approaches have been utilized to elucidate IBD pathogenesis and identify potential biomarkers of disease progression, as reviewed in [126].

Therapeutics used in IBD treatment can influence lipid serum levels, although treatment with biologics, including VDZ, did not result in significant changes compared to baseline 10 weeks after treatment induction [127]. On the other hand, an untargeted serum lipidomics analysis revealed over 100 lipid metabolite features associated with VDZ treatment response, distinguishing between responder and non-responder groups. Significantly for our work, a cluster of these features differed between baseline samples, indicating a possible genetic determinant of VDZ treatment response relating to lipid metabolism pathways. Specifically, six triglycerides were elevated in baseline samples of the responder group, with a characteristic fatty acid composition—all contained a short fatty acid (10–12C), and/or long fatty acid (14, 16, 18C), and almost all contained myristic acid (14C). As a side chain of phorbitol myristate acetate, myristic acid is an immune cell activator and macrophage response regulator [128]. On top of that, N-myristoylation is a co-translational and post-translational modification that primarily serves to promote protein localization to the cell membrane, and is implicated in TLR4 signaling [129]. While we did not identify variants in human N-myrisitoylation genes (hNMT1 and hNMT2) in this patient cohort, further investigation of genes involved in fatty acid metabolism is a potentially lucrative frontier.

There is a lack of studies that examine genomic data in association with VDZ response. One study examining a Saudi Arabian IBD patient cohort (*n* = 16) treated with VDZ showed that the responder group was characterized by variants in genes mediating anti-inflammatory action, such as *CARD9*, *NLRP1*, *IL-4*, and *TYK2*, while the non-responder group was characterized by variants in genes that mediate inflammation, such as *NOD2*, *TRAF1*, *IL10RA*, *IL-23R*, and *IL27* [130]. We found no variants spanning these genes in either the OR or SR group. This discrepancy can most likely be attributed to differences in study design—using results from variant effect prediction models, we excluded all variants that were not classified as damaging from our analysis; also, variant inclusion was independent of locus, while the Saudi Arabian study focused only on those that span IBD-associated loci. Another possibility is that the patient cohorts differed, most likely in disease severity/clinical presentation.

The main limitations of this study include the relatively small sample size, which may reduce statistical power and generalizability; the absence of an independent replication cohort to confirm the observed associations; and the lack of functional validation for the rare damaging variants, leaving their biological consequences uncertain. Although the study is multi-centric and the cohort is ethnically homogeneous, with balanced age and sex distributions between suboptimal and optimal responders, subtle population substructure or other unmeasured demographic factors could still contribute to residual confounding. Therefore, future studies with larger cohorts and integrated functional validation are needed to confirm and extend these results.

## 5. Conclusions

In conclusion, this study underscores the potential of exome-based rare-variant analysis to stratify CD patients and guide precision medicine approaches. The identified genes and pathways may serve as biomarkers for predicting VDZ response or as targets for adjunctive therapies to overcome resistance. Future studies should validate these findings in larger, independent cohorts and evaluate the functional consequences of the implicated variants. Integration with transcriptomic and proteomic data may further clarify how these genetic differences drive distinct cellular and molecular phenotypes in CD.

## Figures and Tables

**Table 1:** Demographic and clinical data of the CD group.

Variable	SR	OR	*p*
Number, counts	31	32	
Male, counts (%)	16 (51.6)	11 (34.4)	0.2 *
Female, counts (%)	15 (48.4)	21 (65.6)	
Age ^#^, years, median (min–max)	59 (23–77)	56.5 (25–79)	0.9 ^†^
Age at diagnosis (Montreal Classification)			
A1 <17 years, counts (%)	0 (0) ^#^	2 (6.3)	0.54 *
A2 17–40 years, counts (%)	12 (40) ^#^	14 (43.7)	
A3 >40 years, counts (%)	18 (60) ^#^	16 (50)	
Disease location (Montreal Classification)			
L1 (terminal ileum), counts (%)	6 (19.4)	7 (21.9)	0.8 *
L2 (colon), counts (%)	11 (35.5)	8 (25)	
L3 (ileum and colon), counts (%)	13 (41.9)	15 (46.8)	
L4 (upper disease), counts (%)	1 (3.2)	2 (6.3)	
Disease behavior (Montreal Classification)			
B1 (inflammatory), counts (%)	19 (61.3)	18 (56.2)	0.72 *
B2 (stricturing), counts (%)	6 (19.4)	10 (31.3)	
B3 (penetrating), counts (%)	5 (16.1)	3 (9.4)	
B1/B2/B3 + perianal disease, counts (%)	1 (3.2)	1 (3.1)	
FC baseline, µg/g, median [IQR]	453 [140–1096]	283 [100–674]	0.11 ^†^
FC 14 week, µg/g, median [IQR]	145 [62.1–342]	100 [31.8–218]	0.14 ^†^
CRP baseline, mg/L, median [IQR]	6.6 [1.85–11.2]	2.5 [0.738–4.68]	0.01 ^†^
CRP 14 week, mg/L, median [IQR]	4.2 [2–7.7]	1.9 [0.975–5.46]	0.04 ^†^
Immunomodulators			
Yes, counts (%)	12 (38.7)	10 (31.3)	0.6 *
No, counts (%)	19 (61.3)	22 (68.7)	
Dose administration at week 10			
Yes, counts (%)	28 (90.3)	28 (87.5)	1 *
No, counts (%)	3 (9.7)	4 (12.5)	

FC—fecal calprotectin, CRP—C-reactive protein, IQR—interquartile range [25th–75th percentile], SR—suboptimal responders, OR—optimal responders, *p*-value of Fisher’s exact test * for discrete or Mann–Whitney test ^†^ for continues variables. In case of Fisher’s exact test, 2 × 2, 3 × 2, and 4 × 2 contingency tables were used depending of the number of groups in categories compared between SR and OR. A *p*-value < 0.05 was considered statistically significant. ^#^—For 1 patient in SR group, age information was missing.

**Table 2:** Variants detected in CD patients with suboptimal and optimal VDZ response.

Variant Type, *n*	SR	OR
Missense all	17,235	17,464
Missense damaging *	549	588
pLoF	345	344
Rare pLoF and missense damaging (MAF < 5% according to gnomAD)	662	691

* Missense damaging variant is defined as avg (REVEL + metaRNN scores) > 0.5; pLoF—predicted loss of function (start/stop lost, stop gained, frameshift, essential splice variants); SR—suboptimal response, OR—optimal response; MAF—minor allele frequency.

## Data Availability

Dataset available on request from the authors.

## Supplementary Materials

The following supporting information can be downloaded at https://www.mdpi.com/article/10.3390/biomedicines14010203/s1, Table S1: Pathways significantly enriched in suboptimal vedolizumab responders; Table S2: Pathways significantly enriched in optimal vedolizumab responders; Table S3: Shared and specific enriched pathways in suboptimal and optimal vedolizumab responders; Table S4: Differences in carrier frequencies of rare variants from enriched pathways between responder groups; Table S5: Differences in the composition of rare variants from enriched pathways between responder groups (SKAT-O analysis); Table S6: Candidate genes linked to suboptimal vedolizumab response (enrichment and carrier burden analysis); Table S7: Candidate genes linked to suboptimal vedolizumab response (SKAT-O analysis); Figure S1: Levels of fecal calprotectin at baseline (week 0) and week 14 of vedolizumab therapy in CD patients. Figure S2: Levels of CRP at baseline (week 0) and week 14 of vedolizumab therapy in CD patients. Figure S3: Heatmap of genes carrying rare damaging variants within enriched Reactome pathways in suboptimal and optimal vedolizumab responders; Figure S4: Heatmap of genes harboring rare damaging variants within pathways identified as significantly different between suboptimal and optimal vedolizumab responders by SKAT-O.
