# Supplementary material for: Pharmacogenomic Pathways Underlying Variable Vedolizumab Response in Crohn’s Disease Patients: A Rare-Variant Analysis
Source: Biomedicines. 2026 Jan 17;14(1):203. doi: 10.3390/biomedicines14010203 (PMC12838795; doi:10.3390/biomedicines14010203)
Supplement: Supplementary file 1 [file biomedicines-14-00203-s001.zip › Stankovic et al. Pharmakogenomics VDZ Supplementary Figures_Proof.pdf.zip!/Stankovic et al. Pharmakogenomics VDZ Supplementary Figures_Proof.pdf]

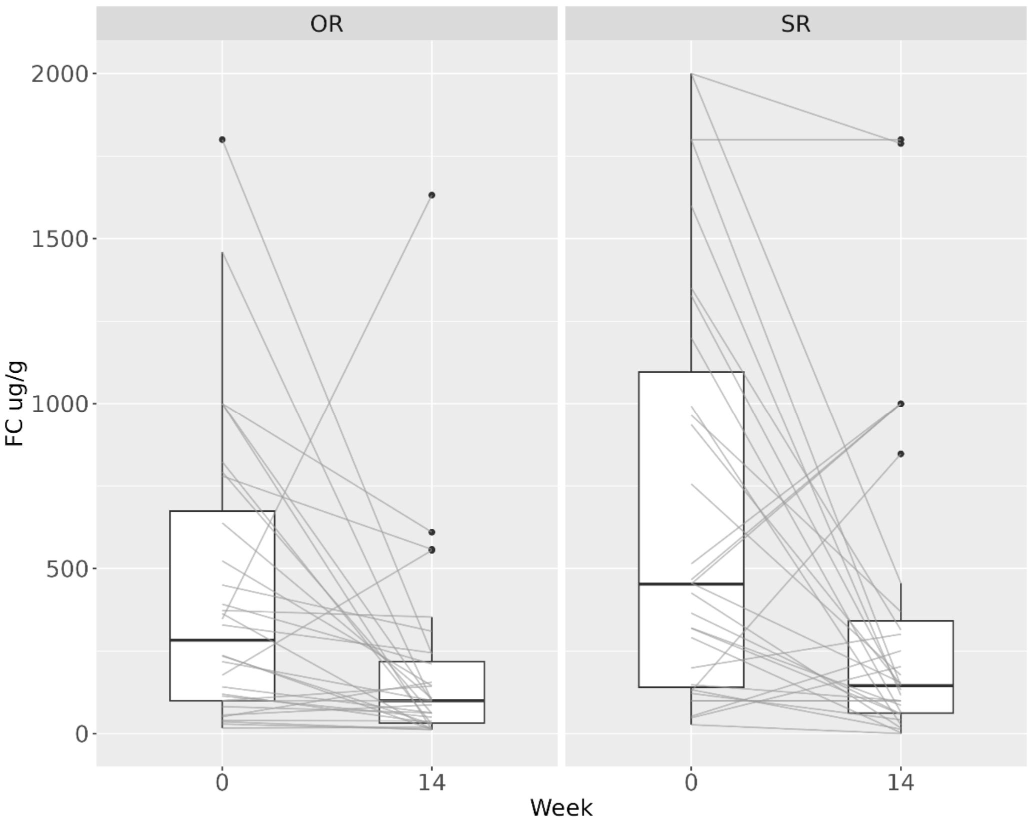

Figure S1. Levels of fecal calprotectin at baseline (week 0) and week 14 of vedolizumab therapy in CD patients. Fecal calprotectin level was significantly decreased at week 14 compared to week 0 in OR ($p = 0.001$) and SR ($p = 0.007$) groups. Difference in fecal calprotectin level between SR and OR in each time point (baseline and week 14) did not reach statistical significance ($p = 0.11$ and $p = 0.14$, respectively). Statistical test performed between time points and within groups was Wilcoxon signed-rank test, while between groups and within time points Mann–Whitney U test. $P < 0.05$ was considered as threshold for statistical significance. OR – optimal responders, SR – suboptimal responders, FC –fecal calprotectin.

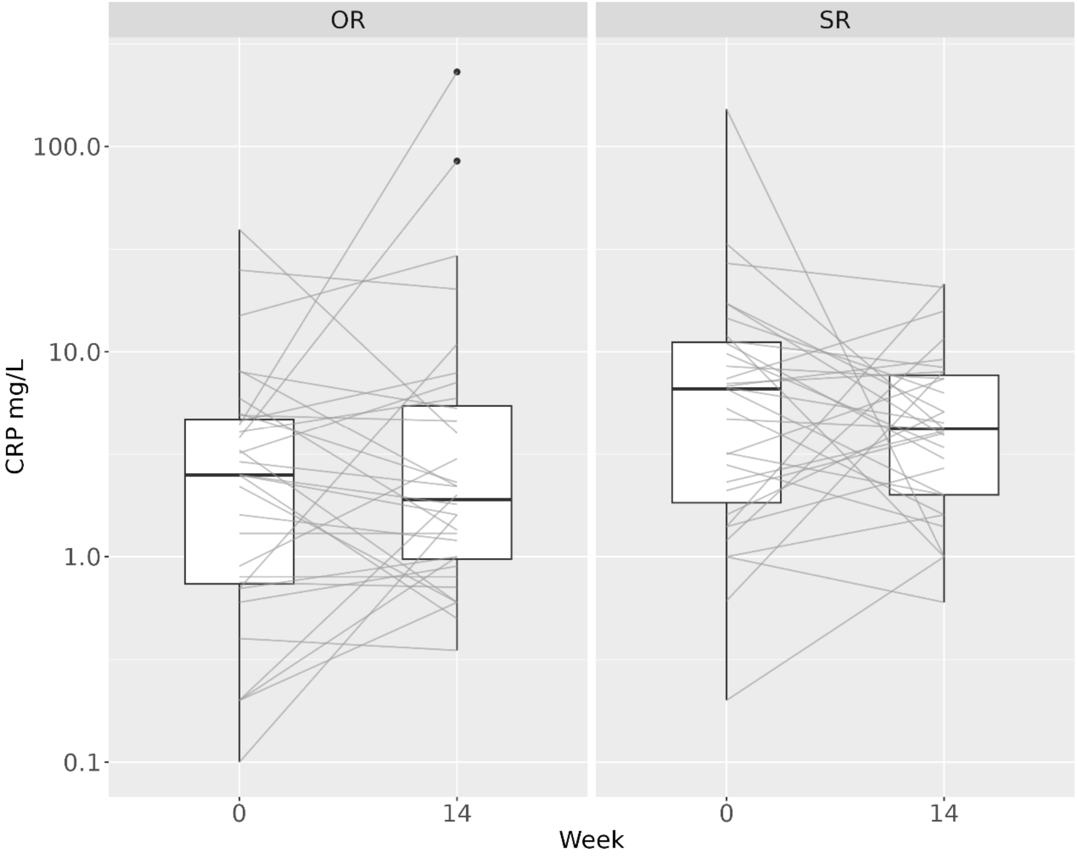

Figure S2. Levels of CRP at baseline (week 0) and week 14 of vedolizumab therapy in CD patients. CRP levels were not significantly different between baseline and week 14 (p = 0.951, p = 0.224, respectively) within each response group. However, CRP level was significantly higher in SR compared to OR group in both week 0 and week 14 (p = 0.01, p = 0.04, respectively). Statistical test performed between time points and within groups was Wilcoxon signed-rank test, while between groups and within time points Mann–Whitney U test. P < 0.05 was considered as threshold for statistical significance. OR – optimal responders, SR –suboptimal responders, CRP – C reactive protein.

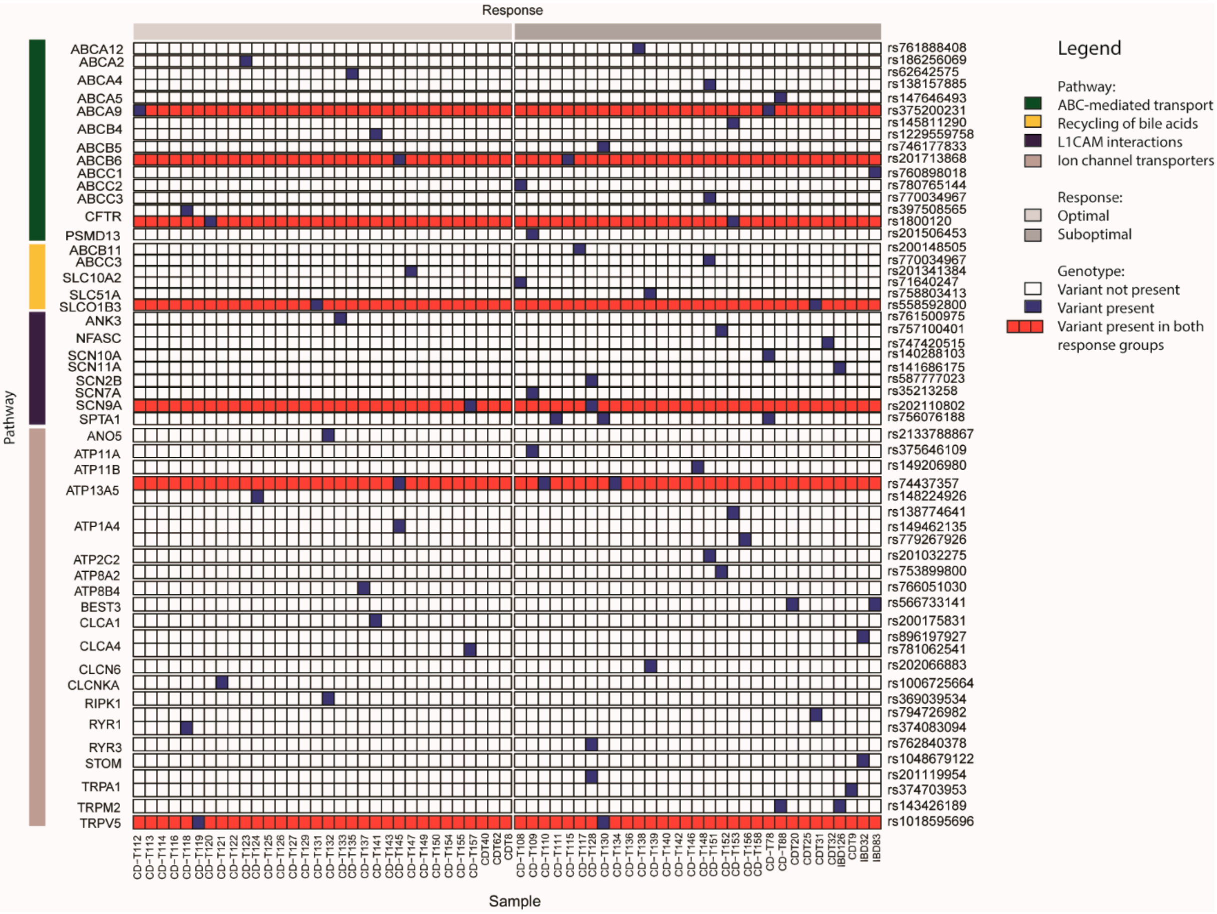

Figure S3. Heatmap of genes carrying rare damaging variants within enriched Reactome pathways in suboptimal and optimal vedolizumab responders. The selected pathways were identified as being specifically associated with suboptimal response through enrichment and carrier burden analyses. The heatmap displays the presence of rare damaging variants in affected genes for each CD patient (white - no rare damaging variant; blue - rare damaging variant present). Rows corresponding to genes that harbor rare damaging variants in both suboptimal and optimal responders are highlighted in red.

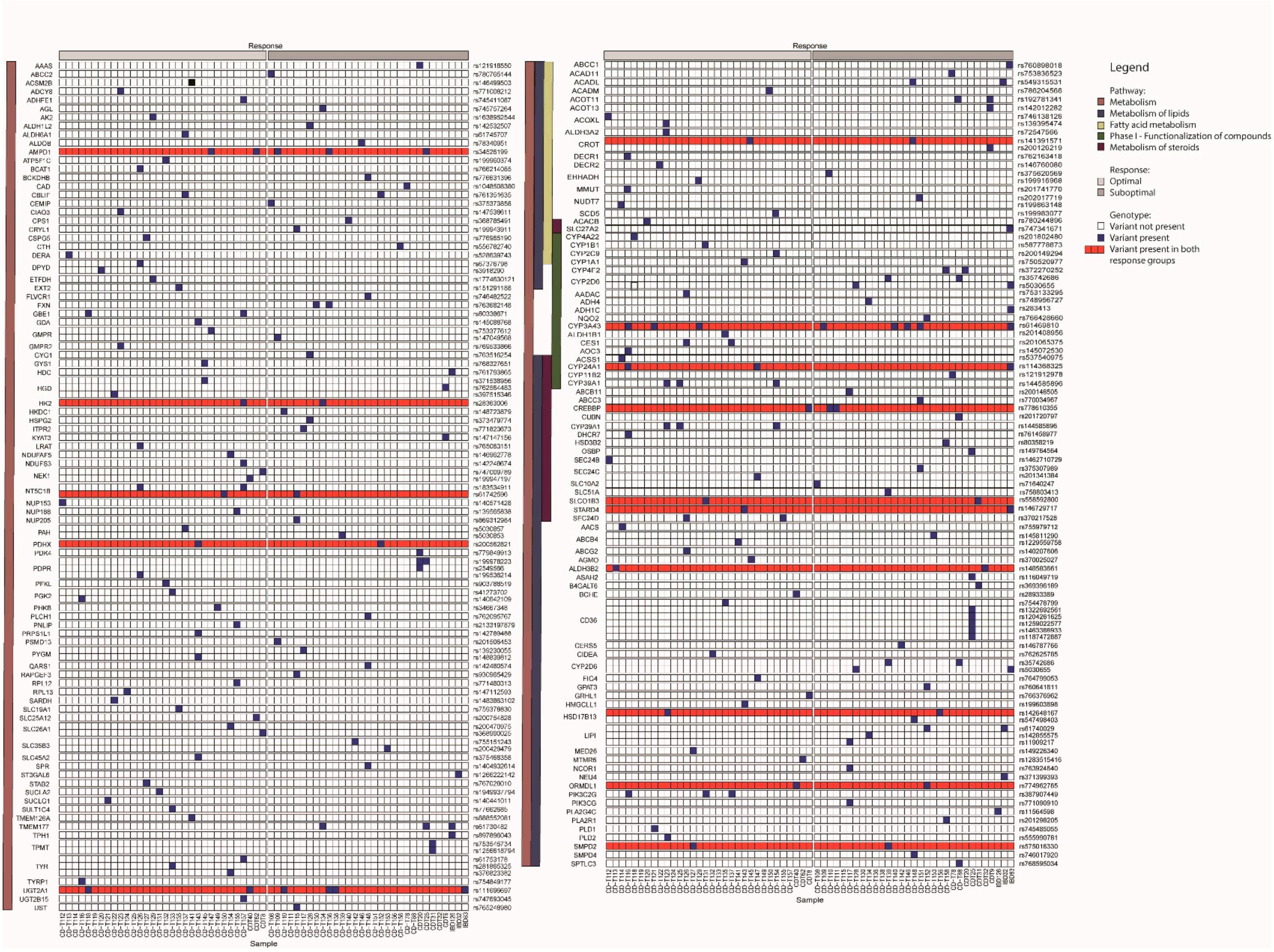

Figure S4. Heatmap of genes harboring rare damaging variants within pathways identified as significantly different between suboptimal and optimal vedolizumab responders by SKAT-O. The heatmap illustrates the distribution of rare damaging variants across affected genes for each CD patient (white - no rare damaging variant; blue - presence of a rare damaging variant). Genes in which rare damaging variants occur in both suboptimal and optimal responders are highlighted in red.
